# Efficacy and safety of phacoemulsification plus goniosynechialysis and trabectome in patients with primary angle-closure glaucoma

**DOI:** 10.1038/s41598-021-92972-9

**Published:** 2021-07-06

**Authors:** Yu Wang, Zhi-qiao Liang, Yu Zhang, Lauren Hennein, Ying Han, Hui-juan Wu

**Affiliations:** 1 Department of Ophthalmology, Peking University People’s Hospital, Eye Diseases and Optometry Institute, Beijing Key Laboratory of Diagnosis and Therapy of Retinal and Choroid Diseases, College of Optometry, Peking University Health Science Center, No. 11 Xizhimen South Street, Xicheng District, Beijing, 100044 China; 2 Department of Ophthalmology, University of California San Francisco, San Francisco, CA USA

**Keywords:** Eye diseases, Prognosis, Therapeutics

## Abstract

We evaluated the efficacy and safety of combined phacoemulsification, intraocular lens implantation, goniosynechialysis (GSL), and trabectome in patients with primary angle-closure glaucoma (PACG). Twenty patients (22 eyes) of PACG treated with combined phacoemulsification, intraocular lens implantation, GSL, and trabectome between September 2017 and September 2020 were included in this case series study. The intraocular pressure (IOP), number of glaucoma medications, and best-corrected visual acuity (BCVA) were recorded at baseline, 1, 3, 6, and 12 months after surgery. Successful surgery was defined as IOP < 21 mmHg with or without IOP-lowering medications. IOP was decreased significantly from 22.07 ± 6.62 mmHg at baseline to 15.06 ± 3.39 mmHg at 12 months’ follow-up (*p *= 0.001). The number of glaucoma medications was significantly reduced from 2.68 ± 1.17 preoperatively to 0.78 ± 0.73 at 12 months’ follow-up (*p *< 0.01). The rate of successful surgery was 88.9% at 12 months. The reduction in IOP showed a positive correlation with baseline IOP (*p *< 0.001), and the reduction in number of glaucoma medications was positively correlated with baseline number of glaucoma medications (*p *< 0.001). There were no vision-threatening complications intraoperatively or postoperatively. Combined phacoemulsification, IOL implantation, GSL, and trabectome were effective and safe in PACG patients in this study. These combined surgical techniques may be useful in PACG patients, especially those with long term and extensive peripheral anterior synechiae.

## Introduction

Primary angle-closure glaucoma (PACG) is a common subtype of glaucoma in China characterized by progressive peripheral anterior synechiae (PAS) that leads to permanent closure of the anterior chamber angle with elevated intraocular pressure (IOP) and subsequent irreversible optic nerve damage^1^. Traditional surgeries for PACG include trabeculectomy and glaucoma valve implantation. Potentially dangerous intraoperative and postoperative complications may occur at a frequency that cannot be ignored, including surgery-related bleb scarring, hypotony, bleb leak, and bleb infection, and these complications are associated with a high rate of additional surgeries^2–4^. Although cataract extraction can deepen a crowded anterior chamber, the closed angle can still remain^5–7^. Combined goniosynechialysis (GSL) with phacoemulsification and intraocular lens (IOL) implantation may fail to achieve a satisfying IOP level secondary to dysfunctional trabecular meshwork (TM)^8–10^. The success rate of combined GSL and cataract surgery is estimated to be between 71.0 and 92.3%^9,11–14^ and subsequent trabeculectomy and glaucoma valve implantation has been shown to be necessary in 2.9–5% patients^15–17^. As a result, minimally invasive glaucoma surgery (MIGS) has been explored in hopes for a more effective and safe alternative.

Trabectome was one of the first-introduced MIGS. Trabectome ablates the TM and inner wall of Schlemm’s canal (SC) to lower IOP, which is the primary structure responsible for outflow resistance in the pathophysiology of glaucoma^18,19^. Considering the need to expose TM tissue during surgery, trabectome was mainly used in various types of open angle glaucoma (OAG). Previous studies investigated that the effectiveness of trabectome in OAG have demonstrated a 22–35% decrease in IOP and a 18–58% decrease in the number of IOP-lowering medications during a follow-up period between 7 and 90 months^18,20–22^.

Since the main resistance of outflow in PACG is located at the obstructed TM tissue, the normal outflow drainage system of SC provides an opportunity to use trabectome in PACG patients after effective GSL. Although previous studies have suggested that the indications of trabectome should be expanded to include narrow angles^23^, to the best of our knowledge, trabectome has not been used for the treatment of PACG. The purpose of this study is to evaluate the efficacy and safety of combined trabectome with phacoemulsification, IOL implantation, and GSL in PACG patients.

## Methods

### Patients

This case series study included 20 patients (22 eyes) diagnosed with PACG and visually significant cataract requiring surgical intervention to control IOP at the Peking University People’s Hospital between September 2017 and September 2020. Six patients were male and 14 were female. All of the 22 eyes received combined phacoemulsification, IOL implantation, GSL, and trabectome. The patients were enrolled in the study when the following baseline criteria were fulfilled: 1. Synechial angle closure greater than 180° as demonstrated on gonioscopy and ultrasound biomicroscopy (UBM); 2. Patients with IOP greater than 21 mmHg or patients with normal IOP with the use of at least one anti-glaucoma medication; 3. The existence of visually significant cataract. Exclusion criteria were as follows: 1. Patients with a history of prior intraocular surgery (except for laser peripheral iridoctomy, laser iridoplasty, and anterior chamber paracentesis); 2. Patients with prior ocular diseases that may affect the anatomy or function of the trabecular meshwork, such as history of ocular trauma, uveitis, and proliferative diabetic retinopathy. Written informed consent was obtained for all patients. Our study followed the guidelines of the Declaration of Helsinki and was approved by the Institutional Review Board of Peking University People’s Hospital (Beijing, China).

### Examination

Before surgery, all patients underwent a comprehensive ophthalmologic examination, including best corrected visual acuity (BCVA), IOP (Goldmann applanation tonometry), and slit lamp examination. Visual acuity was measured with the Snellen visual chart. Visual field perimetry (Swedish Interactive Threshold Algorithm [SITA] 24-2 test of the Humphrey visual field analyzer 750i, Carl Zeiss Meditec, Dublin, CA) was used to evaluate the visual field. Gonioscopy and UBM (Aviso, Quantel Medical, Inc., Bozeman, MT, USA) were performed to determine the extent of PAS. The duration between PACG and surgery was also recorded. PAS was defined as a region of irido-trabecular contact that could not be opened by indentation gonioscopy. Follow-up visits were at the following postoperative periods: 1 day, 1 week, 1 month, 3 months, 6 months, and 12 months. The subsequent long-term follow-up interval was at 1 year. At every visit, BCVA, IOP, intraoperative and postoperative complications, parameters on UBM and number of IOP-lowering medications were recorded. In this study, successful surgery was defined as IOP<21mmHg with or without IOP-lowering medications.

### Surgical procedure: phacoemulsification, IOL implantation, GSL, and trabectome

All surgeries were performed by the same skilled surgeon (HJW) under topical anesthesia. Standard phacoemulsification was performed through a 2.8-mm superior clear corneal incision and an auxiliary temporal corneal paracentesis. After the implantation of a foldable IOL (Model: ZA9003, Johnson & Johnson Surgical Vision, Inc., CA, USA) within the capsular bag, carbachol (1 ml:0.1 mg, Bausch & Lomb , Shandong, CN) was injected into the anterior chamber to induce miosis. GSL using viscoelastic (1ml, Bausch & Lomb, Shandong, CN) was performed circumferentially. With the assistance of an intraoperative surgical gonioscope (Ocular instrument, Inc., USC), after the anterior segment was refilled with viscoelastic, the surgeon gently pressed against the peripheral iris with a blunt cyclodialysis spatula to exert a backwards pressure on the iris and expose the TM at the nasal side. The temporal clear corneal incision was enlarged to 1.7 mm and the trabectome single-use hand piece with the irrigation-aspiration (I/A) system (Neomedix Inc., Tustin, USA) was inserted to reach the nasal anterior chamber. The TM and inner wall of SC were removed about 90°–120° with a power of 0.8 W. Reflux hemorrhage from SC with the withdrawal of the hand piece was a marker of success. Viscoelastic was then removed with the I/A-system.

All eyes were prescribed pilocarpine 2% (5 ml:25 mg, Bausch & Lomb Freda, Shandong, CN) three times per day for the first week after surgery, and then two times per day for four more weeks. Tobramycin and dexamethasone (i.e. Tobradex) eye drops (5 ml, s.a. ALCON-COUVREUR n.v. , Rijksweg 14,B-2870 Puurs, Belgium) were prescribed four times per day or more frequently based on postoperative inflammation and intraocular reflux bleeding. The Tobradex was reduced gradually over several weeks after surgery. IOP-lowering medications were prescribed topically based on postoperative IOP.

### Statistical analysis

The results were analyzed using software SPSS 19.0 (Chicago, USA). All data was calculated as a mean ± standard deviation. The distribution of the data was validated by the Kolmogorov–Smirnov test. The parametric test (Student’s t test) was conducted for some pre- and post-surgical findings. The success of the procedure was analyzed using a Kaplan-Meier analysis. Variables hypothesized to be associated with a reduction in IOP or a reduction in the number of glaucoma medications were analyzed with a univariate linear regression. These variables included age, glaucoma duration, baseline IOP, number of glaucoma medications, and number of clock hours of PAS. A p value of less than 0.05 was considered to be statistically significant.

## Results

Table 1 provides the demographic data of all 20 patients (22 eyes), including six males and fourteen females. All included patients underwent combined phacoemulsification, IOL implantation, GSL, and trabectome surgery. Reflux hemorrhage from SC with the withdrawal of the hand piece was seen in all patients.

**Table 1:** Baseline characteristics of all 20 patients (22 eyes).

Male–female ratio	6:14
Mean age (years)	71.68 ± 6.28 (range 63–85)
Mean glaucoma duration (months)	53.35 ± 58.61 (range 3–192)
Mean follow-up period (months)	10.77 ± 2.72 (range 3–12)
Mean preoperative BCVA	0.38 ± 0.24
Mean preoperative IOP (mmHg)	22.07 ± 6.63
Mean number of preoperative IOP-lowering drugs	2.68 ± 1.17 (range 0–4)
Mean central corneal thickness (μm)	534.87 ± 39.40 (range 483–629)
Mean anterior chanmber depth (mm)	2.41 ± 0.32 (range 1.70–2.88)
Mean axial length (mm)	22.60 ± 0.79 (range 21.63–23.89)
Mean extent of PAS (°)	319.09 ± 64.94 (range 180–360)
Visual field defect (MD)	− 19.14 ± 9.07 (range − 1.02~ to − 30.26)

*BCVA* best corrected visual acuity, *IOP* intraocular pressure, *MD* mean deviation, *PAS* peripheral anterior synechiae.

**Figure 1:**
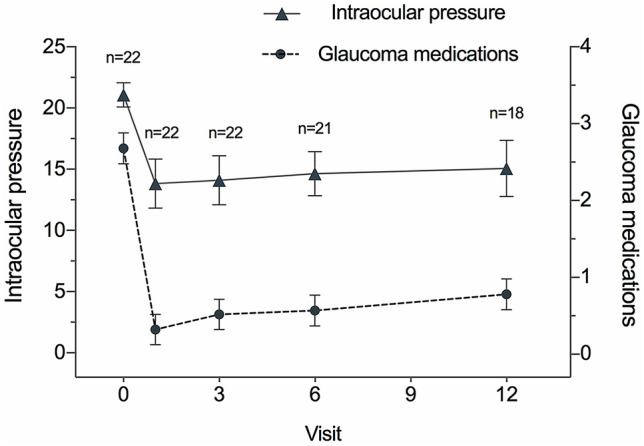
Serial changes of intraocular pressure (IOP) and glaucoma medications from baseline at each follow-up visit. The IOP was significantly decreased at each follow-up visit (1 month: *p* < 0.01; 3 months: *p* < 0.01; 6 months: *p* < 0.01; 12 months: *p* = 0.001, respectively). The IOP-lowering medications was significantly decreased at each follow-up visit (1 month: *p* < 0.01; 3 months: *p* < 0.01; 6 months: *p* < 0.01; 12 months: *p* < 0.01, respectively).

The mean IOP at baseline and postoperatively is demonstrated in Fig. 1. The mean preoperative IOP in patients with PACG was 22.07 ± 6.62 mmHg, which decreased to 15.06 ± 3.39 (− 31.8%) at the mean follow-up period of 10.77 months (*p *= 0.001). Before surgery, patients with PACG used 2.68 ± 1.17 IOP-lowering medications which decreased to 0.78 ± 0.73 (− 70.9%) at 10.77 months’ follow-up (Table 2, Fig. 1) (*p* < 0.01).

**Table 2:** IOP and number of IOP-lowering medications before and after surgery at each follow-up.

	IOP (mmHg)	*p*	Number of medications	*p*
Baseline (n = 22)	22.07 ± 6.62		2.68 ± 1.17	
1 month (n = 22)	13.82 ± 4.11	< 0.01	0.32 ± 0.57	< 0.01
3 months (n = 22)	14.09 ± 3.80	< 0.01	0.52 ± 0.75	< 0.01
6 months (n = 21)	14.64 ± 4.06	< 0.01	0.57 ± 0.68	< 0.01
12 months (n = 18)	15.06 ± 3.39	0.001	0.78 ± 0.73	< 0.01

*IOP* intraocular pressure.

For all patients, the postoperative BCVA was significantly improved compared to baseline (Table 3), with the exception of two eyes whose VA were 0.05 (MD: − 30.26) and light perception respectively with a severe optic nerve damage.

**Table 3:** Best corrected visual acuity before surgery and 1 month after surgery.

	Number				Mean ± SD	*p*
	0–< 0.1	0.1–< 0.3	0.3–< 0.5	≥ 0.5		
Baseline	4	5	3	10	0.39±0.23	
1 month postoperatively	2	4	2	14	0.58±0.33	0.03

*SD* standard deviation.

**Figure 2:**
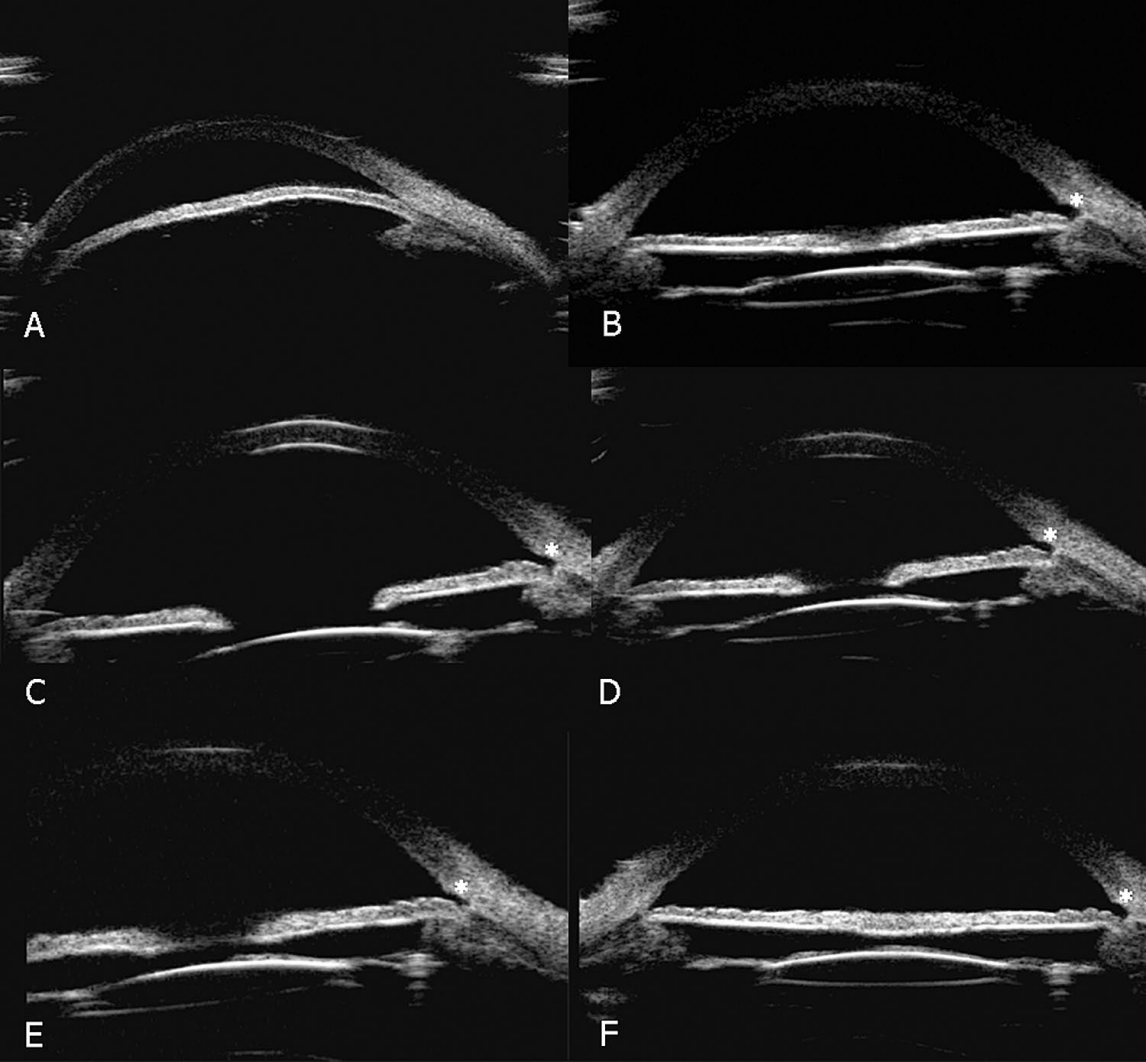
Series ultrasound biomicroscopy (UBM) image of a representative case of primary angle-closure glaucoma after combined phacoemulsification, intraocular lens implantation, goniosynechialysis and trabectome. This representative case was an 81-year-old man with blurred vision for 10 years in the left eye. His best corrected visual acuity (BCVA) was 0.05 and intraocular pressure (IOP) was 20.5 mmHg with the use three anti-glaucoma medications at baseline. (**A**) UBM image preoperatively showed that the anterior chamber angle on the nasal side was closed. (**B**–**F**) UBM image 1 month, 3 months, 6 months, 12 months, and 24 months after surgery demonstrated that the narrow chamber angle had been deepened. At 24 months, the IOP decreased to 14 mmHg with only one medication. White asterisks represent the position that underwent trabectome.

Gonioscopy and UBM revealed that the anterior chamber angle of all 22 eyes was closed to various extents at baseline. Postoperatively, the the anterior chamber angle in all 22 eyes was significantly improved and the position of ablation could be clearly seen at each follow-up. PAS in patients before and after surgery is listed in Table 4. A representative case is shown in Fig. 2.

**Table 4:** Peripheral anterior synechiae in patients before and after operation.

Peripheral anterior synechiae	Open angle	≤ 90°	> 90° ≤ 180°	> 180° ≤ 270°	> 270°
Baseline	0	0	3	4	15
1 month	4	5	3	2	0
3 months	3	6	2	0	0
6 months	6	4	2	0	0
12 months	6	2	1	0	0

Variables hypothesized to be associated with a reduction in IOP and a reduction in the number of glaucoma medications were analyzed with univariate linear regression. The reduction in IOP showed a positive correlation with baseline IOP (p<0.001) and the reduction in the number of glaucoma medications was positively correlated with baseline number of glaucoma medications (*p* < 0.001) (Table 5).

**Table 5:** Univariate regression analysis for factors correlated with the reduction of IOP (R^2^ = 0.649) and reduction of the number of glaucoma medications (R^2 ^= 0.832).

	Reduction of IOP		Reduction of glaucoma medications	
	Univariate coefficient	*p*	Univariate coefficient	*p*
Age	0.127	0.610	− 0.041	0.448
Glaucoma duration	− 0.022	0.386	0.001	0.888
Baseline IOP	0.840	**<** **0.001**	− 0.044	0.385
Baseline number of glaucoma medications	1.612	0.220	1.136	**<** **0.001**
PAS before operation	2.480	0.234	0.222	0.634

Bold values demonstrated the significance of the difference

*IOP* intraocular pressure, *PAS* peripheral anterior synechiae.

Kaplan-Meier analysis showed the rate of successful surgery was 88.9% for PACG at 1 year. No complications occurred intraoperatively. All eyes presented with anterior chamber hemorrhage due to reflux hemorrhage from SC, which was absorbed without additional treatment. Among the 22 eyes, re-occurrence of PAS at the position of the removed TM tissue was observed in only two eyes, which received peripheral argon laser iridoplasty as soon as the reformation of PAS was observed at the 1 month visit. No other postoperative complications occurred.

## Discussion

In this study, we explored the efficacy and safety of trabectome combined with phacoemulsification, IOL implantation, and GSL in PACG patients. We demonstrate that with the assistance of phacoemulsification and re-establishing the anterior chamber angle, trabectome had an effective IOP-lowering effect and was associated with a reduction in the number of IOP-lowering medications at 1 year.

PACG is an ocular disorder characterized by anterior chamber angle closure, which is the main cause of blindness worldwide as well as in China. The primary pathophysiological mechanisms of angle closure include pupillary block and iris plateau configuration. The purpose of treatment is to open the outflow drainage passage and reduce IOP through medical therapy and/or surgery. Phacoemulsification with or without GSL has been shown in many studies to lower IOP in PACG^17,24^. Although phacoemulsification with GSL demonstrate an advantage over traditional surgeries from the point of view of complications, IOP-lowering is not always achieved, especially for patients with a long duration of angle-closure glaucoma^8,9,25^. Tian et al.^25^ compared the efficacy of phacoemulsification combined with GSL in the treatment of acute and chronic angle closure patients. The success rate in the chronic group (acute vs chronic: 100% vs 64.3%) was lower. Lee et al.^8^ compare the effect of phacoemulsification combined with GSL versus phacoemulsification alone in phakic patients with medically well-controlled chronic angle-closure glaucoma. Lee et al. found there was no significant difference between the two groups in IOP reduction and thereby suggested that GSL may not be beneficial. Husain et al.^9^ concluded similar results with Lee et al. through a randomized clinical trial. Furthermore, Wang et al.^10^ reviewed and analyzed seven randomized controlled trials to determine the effect of phacoemulsification and GSL compared to phacoemulsification alone in ACG patients. A “low to moderate quality evidence” was concluded for combined phacoemulsification and GSL surgery when aiming to lower IOP in PACG patients compared to phacoemulsification alone. This indicates that the temporary re-opening of the angle is insufficient in chronic PACG. Even if the angle is kept open for a longer period of time, the re-exposed TM might fail to function. These findings have led to an ongoing effort to explore new strategies that avoid these pitfalls and offer a longer lasting result.

In our study, with the assistance of phacoemulsification and GSL, trabectome offers an effective IOP-lowering effect with a significant reduction in the number of IOP-lowering medications for PACG patients at 1 year. The mean glaucoma duration was 53.35 ± 58.61 months and the mean extent of PAS before operation was 319.09 ± 64.94° in our study. Seventeen eyes had PAS for at least 1 year, and 15 of 22 eyes presented with 360° of synechial angle closure. Previous studies^26,27^ have demonstrated that re-establishing a normal open angle configuration with GSL is not sufficient for successful IOP-lowering. Shihota et al.^28^ proposed that PAS and angle closure causes pigment accumulation and non-inflammatory degeneration of the TM. Konopińska et al.^29^ proposed that the TM is not the only structure that offers outflow resistance of aqueous humor, because 25 and 50% of the resistance also depends on the distal outflow (i.e. collectors channel). For patients with PAS less than 6 months–1 year, TM function can be preserved and the effect of GSL might be sufficient^30,31^. We suggest that the function of the TM and collectors channel of PACG patients with long-term and extensive PAS have undergone irreversible damage. White et al.^32^, Zhao et al.^11^ and Husian et al.^9^ hold a similar opinion, which offers a reasonable explanation as to why the IOP-lowering effect was not always satisfied for patients with a long duration of angle-closure glaucoma.

Another potential limitation of GSL combined with phacoemulsification is that IOP lowering may not be long-lasting due to the reformation of PAS. Lee et al.^8^ reported that postoperative PAS reformation was observed in five patients (33.3%) after GSL. The recurrence of PAS was more extensive compared to preoperatively and the primary site of PAS was identical to that preoperatively. The rate of PAS recurrence was 30–100% after GSL, and the rate was much higher with a longer duration of PAS^8,16,25^. However, only 2 eyes (9%) experienced PAS recurrence in our study. We speculate that trabectome combined with GSL makes the anterior chamber angle wider and also widens the space between the peripheral iris and SC. Even if there is an obvious inflammatory reaction after GSL, PAS may be less likely to reform given this widened space.

Variables hypothesized to be associated with the reduction in IOP and the number of medications were analyzed by univariate regression analysis. We found a greater reduction in IOP and greater reduction in the number of medications in PACG patients who presented with a higher baseline IOP and more IOP-lowering medications respectively. We hypothesized that eyes with higher IOP and more medications may have better results because they may undergo surgery at a shorter interval since diagnosis and thus are less likely to have long-term PAS.

The following study limitations should be considered. First, the sample size was small and the follow-up period was short. Larger-scale, longer-follow up trials are indicated to provide stronger evidence to compare the efficacy of this procedure with that of other operations. Second, we only enrolled PACG patients with concurrent cataract that underwent phacoemulsification, IOL implantation, GSL, and trabectome and thus do not have a control group. Additional studies with two groups who receive phacoemulsification, intraocular IOL and GSL with or without trabectome would be useful to elucidate the efficacy and safety of combined trabectome.

In conclusion, combined phacoemulsification, IOL implantation, GSL, and trabectome is possibly effective and safe to lower IOP and reduce the number of anti-glaucoma medications in PACG patients. This may provide a new method for PACG patients, especially those with long-term and extensive PAS. Further longitudinal studies with a larger sample size and control group are indicated to study this procedure.

## Data Availability

Datasets from the current study are not publicly available due to compliance with patient privacy. Summary statistics are available from the corresponding author on reasonable request.
